# A “Seed-and-Soil” Radiomics Model Predicts Brain Metastasis Development in Lung Cancer: Implications for Risk-Stratified Prophylactic Cranial Irradiation

**DOI:** 10.3390/cancers15010307

**Published:** 2023-01-02

**Authors:** Xiao Chu, Jing Gong, Xi Yang, Jianjiao Ni, Yajia Gu, Zhengfei Zhu

**Affiliations:** 1 Department of Radiation Oncology, Fudan University Shanghai Cancer Center, Shanghai 200032, China; 2 Department of Oncology, Shanghai Medical College, Fudan University, Shanghai 200032, China; 3 Shanghai Clinical Research Center for Radiation Oncology, Shanghai 200032, China; 4 Shanghai Key Laboratory of Radiation Oncology, Shanghai 200032, China; 5 Department of Radiology, Fudan University Shanghai Cancer Center, Shanghai 200032, China

**Keywords:** brain metastasis, non-small cell lung cancer, metastasis prediction, radiomics, prophylactic cranial irradiation, seed-and-soil

## Abstract

**Simple Summary:**

In this proof-of-concept study, we implemented Steven Paget’s “seed-and-soil” theory and proposed that inter-individual differences exist in the brain’s congeniality for developing brain metastasis (BM). Using a non-invasive radiomics method, we demonstrated that a seed-and-soil radiomics model developed from non-metastatic brain magnetic resonance (representing the soil) and primary tumor computed tomography (representing the seed) imaging features can predict BM development in NSCLC patients, which is first-in-class in metastasis prediction studies. Furthermore, a BM radiomics score was developed, and we have shown that this score was significantly correlated with BM-free survival. These results demonstrated that the intrinsic features of a non-metastatic host organ could exert a significant impact on metastasis development, and a host organ’s congeniality for metastasis might be different across individuals, which provides new evidence for the “seed-and-soil” theory and indications for risk-stratified prophylactic cranial irradiation in NSCLC management.

**Abstract:**

Introduction: Brain is a major site of metastasis for lung cancer, and effective therapy for developed brain metastasis (BM) is limited. Prophylactic cranial irradiation (PCI) has been shown to reduce BM rate and improve survival in small cell lung cancer, but this result was not replicated in unselected non-small cell lung cancer (NSCLC) and had the risk of inducing neurocognitive dysfunctions. We aimed to develop a radiomics BM prediction model for BM risk stratification in NSCLC patients. Methods: 256 NSCLC patients with no BM at baseline brain magnetic resonance imaging (MRI) were selected; 128 patients developed BM within three years after diagnosis and 128 remained BM-free. For radiomics analysis, both the BM and non-BM groups were randomly distributed into training and testing datasets at an 70%:30% ratio. Both brain MRI (representing the soil) and chest computed tomography (CT, representing the seed) radiomic features were extracted to develop the BM prediction models. We first developed the radiomic models using the training dataset (89 non-BM and 90 BM cases) and subsequently validated the models in the testing dataset (39 non-BM and 38 BM cases). A radiomics BM score (RadBM score) was generated, and BM-free survival were compared between RadBM score-high and RadBM score-low groups. Results: The radiomics model developed from baseline brain MRI features alone can predict BM development in NSCLC patients. A fusion model integrating brain MRI features with primary tumor CT features (seed-and-soil model) provided synergetic effect and was more efficient in predicting BM (areas under the receiver operating characteristic curve 0.84 (95% confidence interval: 0.80–0.89) and 0.80 (95% confidence interval: 0.71–0.88) in the training and testing datasets, respectively). BM-free survival was significantly shorter in the RadBM score-high group versus the RadBM score-low group (Log-rank, *p* < 0.001). Hazard ratios for BM were 1.056 (95% confidence interval: 1.044–1.068) per 0.01 increment in RadBM score. Cumulative BM rates at three years were 75.8% and 24.2% for the RadBM score-high and RadBM score-low groups, respectively. Only 1.2% (7/565) of the BM lesions were located within the hippocampal avoidance region. Conclusion: The results demonstrated that intrinsic features of a non-metastatic brain exert a significant impact on BM development, which is first-in-class in metastasis prediction studies. A radiomics BM prediction model utilizing both primary tumor and pre-metastatic brain features might provide a useful tool for individualized PCI administration in NSCLC patients more prone to develop BM.

## 1. Introduction

Brain is a major site of metastasis for lung cancer patients, and effective therapy for developed brain metastasis (BM) is limited. Prophylactic cranial irradiation (PCI) has been shown to reduce BM rate and improve survival in small cell lung cancer [1,2], but in non-small cell lung cancer (NSCLC) PCI failed to improve overall survival and had the risk to induce neurocognitive dysfunctions [3,4,5]. This situation indicates that PCI candidates in NSCLC should be carefully selected, yet a promising BM risk stratification method in NSCLC still eludes us.

The “seed-and-soil” theory of metastasis was first proposed by Steven Paget [6], in which he suggested that successful metastasis to distant organs requires not only tumor cells with metastatic potential (the seed), but also congenial host organs (the soil). The seed’s intrinsic properties have been shown to impact various key metastasis processes. On the other hand, although it has long been observed that metastases favor specific organs instead of even distribution across different organs [7], studies focusing on the relationship between the soil’s intrinsic features and metastasis are scarce. Radiomics is a burgeoning field that develops novel biomarkers through quantitative analysis of radiologic images, which reflect the underlying pathophysiologic characteristics of the analyzed regions of interest [8]. Previous studies have shown that radiomics of the primary tumor can predict metastasis in many malignancies, including BM in lung cancer [9,10], which established radiomics evidence for the “seed-and-soil” theory from the seed’s perspective. However, there is no radiomics evidence from the soil’s perspective, as most brain radiomics studies in oncology have only evaluated regions already inflicted with BM [11,12,13]. Considering that radiomics can also extract biological features from non-metastatic organs, we hypothesized that comparing the previously neglected non-metastatic brain imaging of patients who developed BM with those who did not develop BM might reveal the intrinsic features that discern congenial from uncongenial soil.

In this study, we implemented the “seed-and-soil” theory and incorporated the non-metastatic brain radiomic features with primary tumor features to demonstrate whether the combined radiomics model could improve the efficacy of BM prediction, with the goal to risk stratify NSCLC patients and guide individualized PCI administration.

## 2. Methods

### 2.1. Patient Selection

In this study, we screened 1942 pathologically confirmed stage I–IV NSCLC patients who were treated between 2005 and 2020 at our institution (Fudan University Shanghai Cancer Center). A total of 214 patients were finally selected based on the following criteria (detailed in Supplemental Figure S1): have baseline contrast-enhanced chest computed tomography (CT) and brain magnetic resonance imaging (MRI); no evidence of BM before treatment; no other primary malignancies other than NSCLC at baseline and during follow-up; developing BM within three years of follow-up or had no BM confirmed by brain MRI for at least three years.

Patients who developed BM within three years after diagnosis were classified into the BM group, and the others into the non-BM group. We chose three years as the cut-off for BM follow-up because previous studies showed that the curve of cumulative BM incidence for NSCLC patients without baseline BM plateaued at three years after diagnosis [14,15,16,17], indicating that most NSCLC BM develops within three-year follow-up. Among the 256 patients included, there were 128 in the BM and 128 in the non-BM group. The detailed patients’ clinical characteristics are presented in Table 1. The median follow-up was 52 (range: 36 to 84) months for the non-BM cohort and 20 (range: 1 to 73) months for the BM cohort. The median time interval from initial diagnosis to BM was 17 (range: 1 to 36) months. The study was approved by the institutional ethics board of Fudan University Shanghai Cancer Center (approval code SCCIRB2021130-1). Individual consent for this retrospective study was waived.

### 2.2. Radiomics Model Development

For radiomics analysis, both the BM and non-BM groups were randomly distributed into training and testing datasets at a 70%:30% ratio. The training dataset included 89 non-BM and 90 BM cases, whereas the testing dataset included 39 non-BM and 38 BM cases. We first developed radiomic models using the training datasets and subsequently validated the models in the corresponding testing datasets.

To develop the brain MRI radiomic model, the entire brain region in the T1-weighted (T1w) image of the baseline brain MRI was extracted via a deep learning algorithm. A publicly available Python package named “DeepBrain” was employed to build the segmentation model (https://github.com/iitzco/deepbrain, accessed on 9 August 2022). Then, the 3D brain regions were segmented on T1w images by using the pre-trained U-Net model in the “DeepBrain” package. To decode the imaging phenotypes of BM, a set of 1046 radiomic features was computed based on the segmented T1w brain images. The brain radiomic features were composed of three types of imaging features, namely, original image feature, Laplace of Gaussian image feature, and wavelet image feature [18]. Each of these three types of imaging features contained shape features, intensity features (first-order features), and texture features.

To develop the chest CT imaging feature-based radiomics model, the chest CT scans of primary lung tumor were used to compute the radiomic features [19]. The primary tumors were delineated by a radiation oncologist (X.C.) and reviewed by a senior radiologist (Y.G.) and a senior radiation oncologist (Z.Z.) in a slice-by-slice fashion. Then, 1118 radiomic features were calculated based on the segmented primary tumor to decode the imaging heterogeneity of the primary lung tumor.

**Figure 1:**
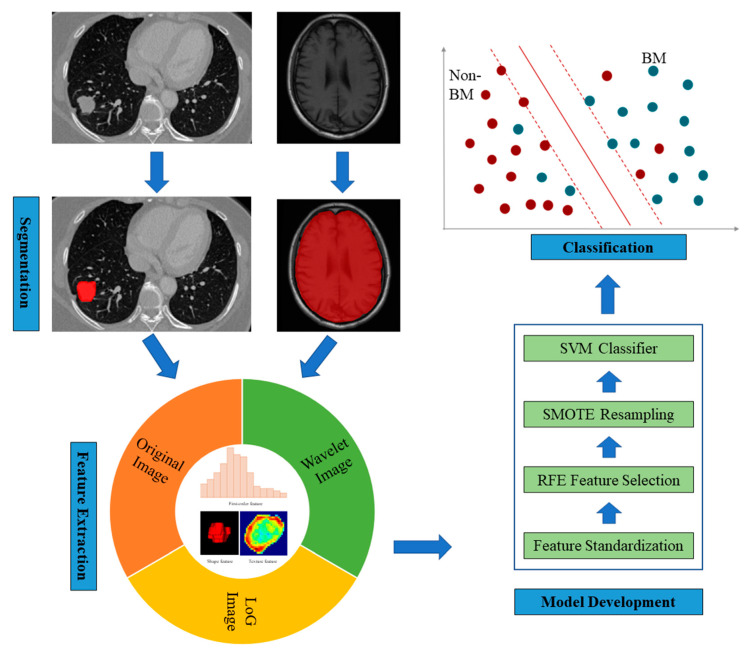
Illustration of the radiomics model development procedures.

After extracting the radiomic features of the primary tumor and the pre-metastatic brain, a standard scaler was applied to standardize each imaging feature by removing the mean and scaling to unit variance. To reduce the dimensionality of MRI and CT image features, a recursive feature elimination (RFE) feature selection method was used to remove redundant features. The RFE feature selector was configured with a linear support vector machine (SVM) estimator. Given that the number of BM and non-BM patients in the training dataset was imbalanced, a synthetic minority oversampling technique was used to resample the training dataset by increasing the minority cases [20]. Finally, an SVM classifier was applied to build a prediction model to classify between BM and non-BM patients. The MRI radiomics model and CT radiomics model were developed by using the same feature processing technique and SVM classifier. To further improve the model performance, a score fusion strategy was applied to fuse the prediction scores generated by the MRI radiomics model and the CT radiomics model. In this study, the prediction scores of two models were fused with weighted strategy, minimum fusion strategy, and maximum fusion strategy. The weighted strategy was performed by increasing weighted factor of the prediction score generated by the MRI radiomics model from 0.1 to 0.9 (or systematically decreasing the weighting factor from 0.9 to 0.1 applied to the prediction scores generated by CT radiomics model). The minimum fusion strategy and maximum fusion strategy were performed by choosing the maximum or minimum of the prediction scores generated by the two models. The radiomics model development procedures are illustrated in Figure 1.

To evaluate the performance of our proposed radiomics model, the area under the receiver operating characteristic (ROC) curve (AUC) and the corresponding 95% confidence interval (CI) were computed. A bootstrap resampling procedure with 1000 iterations was applied to estimate the 95% CI. The DeLong test was employed to compare the ROCs of different radiomic models. Moreover, several quantitative metrics, namely, accuracy (ACC), sensitivity, specificity, positive predictive value (PPV), negative predictive value (NPV), and odds ratio (OR), were calculated to assess the performance of our proposed radiomics model. To obtain a binary classification result, the Youden index-based cut-off of radiomics score was used to divide the patients into high- and low-risk groups for BM.

All the model development and performance evaluation processes were implemented on Python programming software, with several publicly available packages, namely, SimpleITK, Pyradiomics, Scikit-learn, SciPy, Matplotlib, NumPy, and Pandas.

### 2.3. Documentation of the NSCLC Brain Metastasis Lesions

To evaluate the feasibility of hippocampus-avoidance (HA) PCI, the hippocampi were delineated on MRI images in the patients from the BM group, according to Radiation Therapy Oncology Group 0933 protocol [21]. All BM lesions were documented according to their central locations from the hippocampus. Hippocampal metastasis was defined as the lesion’s center located within 5 mm of the hippocampus.

### 2.4. Statistics

Continuous variables were summarized by descriptive statistics, such as standard deviations, medians, and ranges. Categorical variables were tabulated by frequency and percentage. The prognostic significance of RadBM score was evaluated via Cox proportional-hazards regression. Kaplan–Meier plots were used to visualize the event-time distributions in survival analysis, and differences between groups were compared via log-rank test. All statistical tests were two-sided, and *P* values of less than 0.05 were considered statistically significant. Statistical Package for Social Sciences (SPSS version 20.0, IBM, New York, NY, USA) software was used for all statistical analyses. Kaplan–Meier plots were generated via GraphPad Prism 9 (GraphPad Software, La Jolla, CA, USA).

## 3. Results

**Figure 2:**
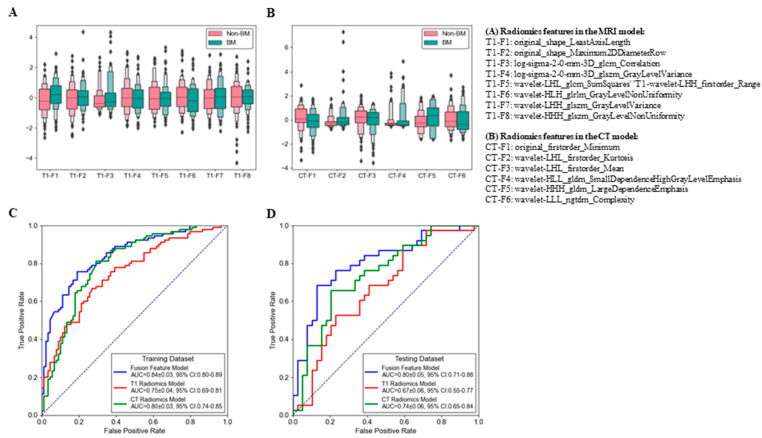
Radiomic features and brain metastasis prediction efficacy of the radiomic models. (**A,B**) Boxplots of the selected radiomic features in (**A**) the brain MRI model, (**B**) chest CT model. (**C,D**) Receiver operating characteristic curves of the radiomic prediction model in the training (**C**) and testing (**D**) datasets.

Figure 2 illustrates the boxplots of the selected radiomic features in the brain MRI model, the chest CT model, and the fusion feature model representing the brain MRI and chest CT radiomic features. A total of eight radiomic features were selected in the brain MRI radiomics model (Figure 2A), and six features were selected in the CT radiomics model (Figure 2B). 

Both BM prediction models developed from the CT and MRI radiomic features showed acceptable predictive efficacy. The MRI radiomics model displayed AUCs of 0.75 (95% confidence interval (CI): 0.69–0.81; Figure 2C) and 0.67 (95% CI: 0.55–0.77; Figure 2D) in the training and testing datasets, respectively. The CT radiomics model displayed AUCs of 0.80 (95% CI: 0.74–0.85; Figure 2C) and 0.74 (95% CI: 0.65–0.84; Figure 2D) in the training and testing datasets, respectively. 

Referring to the “seed-and-soil” theory, we hypothesized that combining brain MRI (the soil) and primary tumor CT (the seed) radiomic features may provide complementary information in predicting BM. To validate our hypothesis, a fusion radiomics model was developed by incorporating brain MRI and primary tumor CT radiomic features. As expected, the “seed-and-soil” fusion radiomics model displayed improved predictive efficacy with AUCs of 0.84 (95% CI: 0.80–0.89; Figure 2C) and 0.80 (95% CI: 0.71–0.88; Figure 2D) in the training and testing datasets, respectively. The predictive efficacy of this fusion model was significantly better than that of the CT model (*p* = 0.001 and 0.008 for the training and testing datasets, respectively) or MRI model (*p* = 0.004 and 0.010 for the training and testing datasets, respectively). The ACC, sensitivity, specificity, PPV, NPV, and odds ratio of the radiomic models are summarized in Table 2.

**Figure 3:**
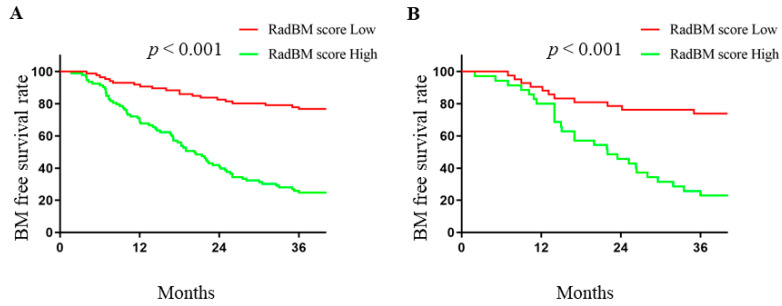
Kaplan–Meier curves of brain metastasis-free survival according to RadBM score in the training (**A**) and testing (**B**) datasets. Comparison between groups were calculated using Log-rank test.

A RadBM score was constructed from the seed-and-soil radiomics model and patients were allocated to RadBM score-high and RadBM score-low groups according to the median of the RadBM score (range: 0.029–0.924, median: 0.387). BM-free survival was significantly shorter in the RadBM score-high group versus the RadBM score-low group in both the training dataset (Log-rank *p* < 0.001, Figure 3A) and the testing dataset (Log-rank *p* < 0.001, Figure 3B). Hazard ratios for BM were 1.056 (95% CI: 1.042–1.069) and 1.062 (95% CI: 1.034–1.092) per 0.01 increment in RadBM score in the training dataset and testing dataset, respectively. Cumulative BM rate at three years were 75.8% and 24.2% for the RadBM score-high and RadBM score-low groups, respectively.

### NSCLC Brain Lesion Distribution and Implication for Hippocampal Avoidance PCI

The distribution of NSCLC BM lesions in 128 patients from the BM group are shown in Table 3. There was a total of 565 BM lesions; all lesions were categorized according to their central locations from the hippocampi. Among them, only 7 lesions were located in the HA region (1.2%), and there were 23 lesions located within the 5–15 mm zone adjacent to the hippocampi (4.1%), while the remaining 535 lesions were located at least 15 mm away from the hippocampi (94.7%, Table 3).

## 4. Discussion

Lung cancer is one of the brain-favoring malignancies when metastasis develops. The incidence of BM in NSCLC is around 30%, and the brain had been the initial site of recurrence in 15% to 40% of cases [22,23,24,25,26,27]. However, despite its success achieved in SCLC, PCI failed to improve overall survival and increased the risk of neurocognitive dysfunctions [3,4,5]. These results suggest that PCI candidates in NSCLC should be carefully selected, which requires an efficient BM risk stratification tool to identify patients at high risk of BM.

It has long been accepted that cooperation between tumor cells (the seed) and the host organ (the soil) is crucial for successful tumor metastasis. Abundant evidence has demonstrated that different tumors display specific organ-tropism during metastasis, supporting the theory of seed-and-soil interaction [28,29]. However, evidence for discerning inter-individual differences in the congeniality of the non-metastatic human brain remains lacking, because it would be impractical to obtain tissues from a non-metastatic organ. In this proof-of-concept study, we demonstrated for the first time that the intrinsic features of the non-metastatic human brain alone could exerted a significant impact on BM development. The results from this study also revealed that a radiomics BM prediction model could be a promising tool for screening NSCLC patients for BM prevention.

Using a non-invasive radiomics method, we showed that a model developed from non-metastatic host organ radiomic features can predict metastasis to this organ, which is first-in-class in metastasis prediction studies. Furthermore, the fusion “seed-and-soil” radiomics model integrating brain radiomic features with primary tumor radiomic features provided a synergetic effect in predicting BM, with AUCs of 0.84 and 0.80 in the training and testing datasets, respectively. This model is significantly more efficient than the brain MRI model and the chest CT model. Moreover, a RadBM score developed from the “seed-and-soil” radiomics model could effectively discern high BM risk NSCLC cases from lower risk cases, which should be spared the PCI for better preservation of neurocognitive functions. Furthermore, the HA-PCI technique, a novel PCI delivery method utilizing the modern intensity-modulated radiotherapy technique to avoid conformally the hippocampal neural stem-cell niche were developed to better preserve the neurocognitive functions during cranial irradiation [30,31,32,33]. A major concern of employing HA-PCI is the risk of developing BM in the HA region. We evaluated the distribution of NSCLC BM lesions and their location relative to the hippocampi and found a 1.2% rate of HA region BM development (Table 3); thus, the risk of BM development in the HA region is relatively low and it would be safe to employ the HA technique in our NSCLC cohort.

As a result of rapidly expanding therapies, we are expecting longer survival and more metastasis development in NSCLC patients. An integrated risk prediction model taking both the seed’s and the soil’s features into account may prove useful in guiding clinical practice (e.g., more intense surveillance or PCI for high-risk patients). The evidence provided in this study has also consolidated the theory that the “soil” plays a crucial role in BM development independent of the “seed”. We have also verified that the HA technique is safe because the majority of BM lesions recorded in our NSCLC cohort were located outside the HA region.

## 5. Conclusions

In conclusion, we demonstrated that by employing the seed-and-soil radiomics BM prediction model, it is possible to discern high BM risk patients (those who benefit most from BM prevention PCI) from the low BM risk patients (those who should not receive PCI to prevent unnecessary neurocognitive toxicities). However, the advantages and disadvantages of PCI in NSCLC should be weighed cautiously in the setting of a prospective randomized trial utilizing both the BM risk stratification tool and the HA-PCI technique before a conclusion can be drawn.

## Figures and Tables

**Table 1:** Patients’ clinical characteristics.

Characteristic	BM + Cohort (*n* = 128)	BM − Cohort (*n* = 128)	*p*
Gender	☐	☐	0.676
Male	66	56	
Female	62	72	
Smoking status	☐	☐	0.52
ever-smokers	52	46	
never-smokers	76	82	
TNM stage	☐	☐	0.566
I	32	45	
II	37	29	
III	7	6	
IV	52	48	
Age at diagnosis (years)	57 (30–72)	60 (32–76)	<0.001
Follow-up (months)	17 (1–73)	52 (36–84)	<0.001

Staging was based on the 8th TNM staging system for lung cancer.

**Table 2:** Brain metastasis prediction efficacy of the radiomic models.

Model		ACC (%)	Sensitivity (%)	Specificity (%)	PPV (%)	NPV (%)	OR
MRI radiomics model	Training dataset	71.3	75.5	65.2	76.2	64.3	5.8
	Testing dataset	60.5	38.5	94.1	90.9	50	10
CT radiomics model	Training dataset	73.7	87.3	53.6	73.6	74	7.9
	Testing dataset	67.4	50	94.1	92.9	55.2	16
“Seed-Soil” fusion radiomics model	Training dataset	77.7	74.4	80.9	79.8	75.8	12.3
	Testing dataset	71.4	78.9	64.1	68.2	75.8	6.7

ACC: accuracy; PPV: positive predictive value; NPV: negative predictive value; OR: odds ratio.

**Table 3:** Distribution of Brain Metastasis in NSCLC patients.

	Within HA	Rest of The Brain	
	within 5 mm of HC	5–15 mm from HC	>15 mm from HC
No. of lesions (565 in 128 patients)	7	23	535

Abbreviation: NSCLC, non-small cell lung cancer; HA: hippocampal avoidance region; HC: hippocampi.

## Data Availability

The data presented in this study are available upon request, please contact the corresponding authors.

## Supplementary Materials

The following supporting information can be downloaded at: https://www.mdpi.com/article/10.3390/cancers15010307/s1, Figure S1: Patient selection workflow.
