# Supplementary material for: A “Seed-and-Soil” Radiomics Model Predicts Brain Metastasis Development in Lung Cancer: Implications for Risk-Stratified Prophylactic Cranial Irradiation
Source: Cancers (Basel). 2023 Jan 2;15(1):307. doi: 10.3390/cancers15010307 (PMC9818608; doi:10.3390/cancers15010307)
Supplement: Supplementary file 1 [file cancers-15-00307-s001.zip › cancers-2122142-supplementary.pdf]

cancers

MDPI

Article

A “Seed-and-Soil” Radiomics Model Predicts Brain Metastasis Development in Lung Cancer: Implications for Risk-Stratified Prophylactic Cranial Irradiation

Xiao Chu, Jing Gong, Xi Yang, Jianjiao Ni, Yajia Gu and Zhengfei Zhu

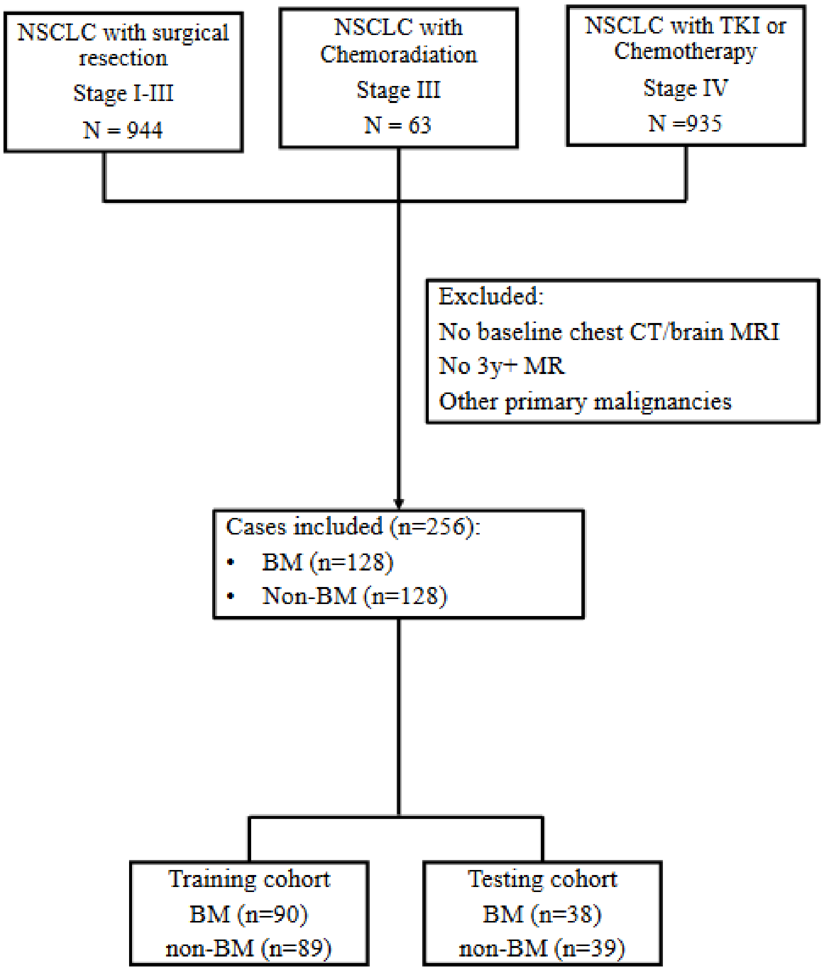

Figure S1. Patient selection workflow.
